# Longitudinal trajectories of pneumonia lesions and lymphocyte counts associated with disease severity among convalescent COVID-19 patients: a group-based multi-trajectory analysis

**DOI:** 10.1186/s12890-021-01592-6

**Published:** 2021-07-13

**Authors:** Nannan Shi, Chao Huang, Qi Zhang, Chunzi Shi, Fengjun Liu, Fengxiang Song, Qinguo Hou, Jie Shen, Fei Shan, Xiaoming Su, Cheng Liu, Zhiyong Zhang, Lei Shi, Yuxin Shi

**Affiliations:** 1 Department of Radiology, Shanghai Public Health Clinical Center, Fudan University, Shanghai, 201508 China; 2 Shanghai Key Laboratory of Artificial Intelligence for Medical Image and Knowledge Graph, Shanghai, 200051 China; 3 Institute of Healthcare Research, Yizhi, Shanghai, China; 4 Shanghai Institute for Advanced Communication and Data Science, Shanghai University, Shanghai, China; 5 School of Communication and Information Engineering, Shanghai University, Shanghai, China; 6 Fudan University, Shanghai, China

**Keywords:** COVID-19, Group-based multi-trajectory modelling, Clinical course, Pneumonia, Lymphocyte

## Abstract

**Background:**

To explore the long-term trajectories considering pneumonia volumes and lymphocyte counts with individual data in COVID-19.

**Methods:**

A cohort of 257 convalescent COVID-19 patients (131 male and 126 females) were included. Group-based multi-trajectory modelling was applied to identify different trajectories in terms of pneumonia lesion percentage and lymphocyte counts covering the time from onset to post-discharge follow-ups. We studied the basic characteristics and disease severity associated with the trajectories.

**Results:**

We characterised four distinct trajectory subgroups. (1) Group 1 (13.9%), pneumonia increased until a peak lesion percentage of 1.9% (IQR 0.7–4.4) before absorption. The slightly decreased lymphocyte rapidly recovered to the top half of the normal range. (2) Group 2 (44.7%), the peak lesion percentage was 7.2% (IQR 3.2–12.7). The abnormal lymphocyte count restored to normal soon. (3) Group 3 (26.0%), the peak lesion percentage reached 14.2% (IQR 8.5–19.8). The lymphocytes continuously dropped to 0.75 × 10^9^/L after one day post-onset before slowly recovering. (4) Group 4 (15.4%), the peak lesion percentage reached 41.4% (IQR 34.8–47.9), much higher than other groups. Lymphopenia was aggravated until the lymphocytes declined to 0.80 × 10^9^/L on the fourth day and slowly recovered later. Patients in the higher order groups were older and more likely to have hypertension and diabetes (all P values < 0.05), and have more severe disease.

**Conclusions:**

Our findings provide new insights to understand the heterogeneous natural courses of COVID-19 patients and the associations of distinct trajectories with disease severity, which is essential to improve the early risk assessment, patient monitoring, and follow-up schedule.

**Supplementary Information:**

The online version contains supplementary material available at 10.1186/s12890-021-01592-6.

## Background

The ongoing coronavirus disease 2019 (COVID-19) pandemic poses unprecedented challenges to healthcare systems worldwide. Medical scientists have claimed that it is urgent to understand the complete natural course of COVID-19 to optimise follow-up strategies to improve patient outcomes such as minimising persistent sequelae [1]. To date, several related studies were published [2–7], which, however, were limited for the following reason. The population-level averages of biomarkers across a certain time interval were computed and the estimations of successive intervals over time were connected to outline the course. Such an approach mixed all patients together and could not differentiate the heterogeneity between patients who had distinct disease severity, clinical progression, and long-term prognosis [5, 8].

Group-based multi-trajectory modelling (GBMTM) is a latent class technique that takes full advantage of multivariate longitudinal data that are interrelated and complementary indicators of disease progression [9]. GBMTM decomposes the population into distinct groups, each with a different underlying trajectory in terms of the studied biomarkers, therefore is suitable for capturing the longitudinal characteristics of COVID-19.

CT has proven to be a diagnostic and follow-up tool of high sensitivity. The dynamic changes of percentage of lesion volume is an intuitive metric to quantitatively assess the evolution of pneumonia and evaluate the changes of pneumonia severity degrees [10–15]. Moreover, one recent observational study described ex-COVID-19 patients at an average of three months after the infection, and it showed that while the pulmonary parenchyma markedly recovered, residual abnormalities were frequently existed and were associated with lower lung function [16].

Several studies have investigated measurements of laboratory parameters and their correlation with CT as a way to prognosticate infection severity and convalescence, and the involved laboratory parameters include C-reactive protein (CRP), lactate dehydrogenase (LDH), and erythrocyte sedimentation rate (ESR) [17, 18]. Mandal’s report found that despite significant abnormalities at discharge, blood test results had returned to normal levels in the majority of patients at follow-up. However, 7.3% of 247 patients had persisting lymphopaenia [19]. In this study, we investigate one of the easily obtained predictors, namely the lymphocyte count. Lymphocytes play an important role in maintaining the immune system function. Decreasing lymphocytes indicate that a number of immune cells are damaged and immune function is impaired. Emerging research suggested that the evaluation of functional activity of lymphocytes in recovered individuals could make a huge contribution in attempts to characterize the protective immune response against SARS-CoV-2 infection [20–22]. Both abnormal imaging results and decreased lymphocytes are typical characteristics commonly observed among COVID-19 patients and longitudinal tracing data could be available across the clinical course [23].

The primary goal of this study was applying GBMTM to identify distinct longitudinal trajectories in terms of pneumonia lesion percentage and lymphocyte count to efficiently and transparently assess the dynamic nature of the disease course starting from the symptom onset to post-discharge among various COVID-19 patients. The identified trajectories could be used to subtype patients for further studies and provide guidance for optimising follow-up schedules on a case-by-case basis.

## Methods

### Ethics statement

This retrospective study was approved by the Ethics Committee of Shanghai Public Health Clinical Center (SPHCC) (YJ-2020-S035-01) and was carried out in adherence with the Declaration of Helsinki. The need for informed consent from all patients was waived due to the study’s retrospective nature.

### Patients

**Fig. 1:**
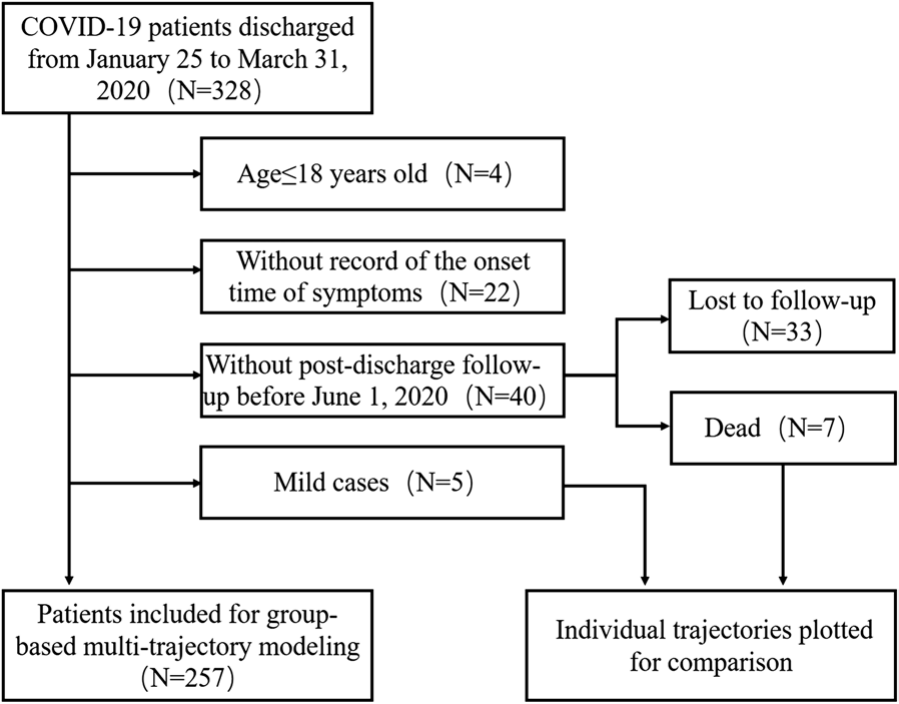
Patient selection flowchart

SPHCC is the only designated hospital for COVID-19 in Shanghai, with the first case reported on January 20, 2020. By March 31, 2020, 328 laboratory-confirmed patients were discharged and were initially eligible for our research. Inclusion criteria were applied: (1) age ≥ 18 years old; (2) reported history of symptoms and the date of initial symptoms; (3) with at least one post-discharge follow-up. One exclusion criterion was: diagnosed as mild cases with no pneumonia lesions on CT scans throughout hospitalisation. As a result, a cohort of 257 COVID-19 patients (131 male and 126 females) were included for GBMTM trajectory analysis (Fig. 1), and they were followed up after discharge until June 1, 2020 with clinical symptoms monitored and blood tests and CT scans conducted as needed. For the sake of research integrity, we further investigated the mild (N = 5) and fatal cases (N = 7) for comparison.

### Data extraction

Clinical data were obtained from the electronic medical record system, including sociodemographic characteristics (for example, age and sex), clinical characteristics (for example, the date of initial symptom onset, date of discharge, clinical subtype on admission, worst clinical subtype during hospitalisation, symptoms, and comorbidity) and longitudinal lymphocyte count records. Lymphopenia was defined as lymphocyte count below 1.1 × 10^9^/L. COVID-19 patients were categorized into mild, moderate, severe, and critical cases according to the Chinese national guidelines [24, 25]. Chest CT acquisition and automatic COVID-19 pneumonia lesion quantification based on artificial intelligence are detailed in the supplementary materials.

### Group-based multi-trajectory modelling

We applied GBMTM to identify subgroups of COVID-19 who had distinct longitudinal trajectories with respect to lesion percentage and lymphocyte count routinely collected from onset to post-discharge. Time 0 of the trajectory was the date of initial symptom onset. GBMTM analyses were introduced in detail in the supplementary materials.

### Statistical analysis

Continuous data were expressed as median (interquartile range [IQR]), and categorical data were denoted as count (percentage). Data between trajectory groups were compared with the Kruskal-Wallis, chi-squared, and Fisher’s exact tests as appropriate. A two-tailed P value less than 0.05 was considered statistically significant. All of the analyses were conducted using R software version 3.6.2 (R Foundation for Statistical Computing, Vienna, Austria).

## Results

### Patient characteristics

The median age of the 257 patients included in the GBMTM analysis was 51 years (IQR 37–63), 131 were male (51.0%), and 20 were smokers (7.8%) (Table 1). The most common symptoms were fever (83.3%) and cough (81.7%). Most patients were moderate cases on admission (97.3%) and during hospitalisation (94.2%). At admission, only 6 patients were severe or critical cases, but this number increased to 15 during hospitalisation. A total of 1,815 CT scans and 1,776 lymphocyte count records were collected. The median numbers of CT scans and lymphocyte data points per patient were 7 (IQR 6–8) and 6 (IQR 5–8), respectively. Of note, we also analysed 5 mild cases and 7 fatal cases for comparison.

**Table 1:** Clinical characteristics of the 257 included patients

Characteristics	Value
Age, years (IQR)	51 (37, 63)
Male sex, n (%)	131 (51.0%)
Smoking, n (%)	20 (7.8%)
*Symptoms*	
Fever	214 (83.3%)
Cough	210 (81.7%)
Diarrhoea	49 (19.1%)
*Clinical subtype on admission*	
Mild	1 (0.4%)
Moderate	250 (97.3%)
Severe	5 (1.9%)
Critical	1 (0.4%)
*Worst clinical subtype during hospitalisation*	
Moderate	242 (94.2%)
Severe	10 (3.9%)
Critical	5 (1.9%)
*Comorbidity*	
Cardiovascular disease	18 (7.0%)
Hypertension	59 (23.0%)
Coronary heart disease	18 (7.0%)
Diabetes	27 (10.5%)
Chronic obstructive pulmonary disease	6 (2.3%)
Cancer	6 (2.3%)
Number of CT scans per patient	7 (6, 8)
Number of lymphocyte count tests per patient	6 (5, 8)

Statistics presented: median (IQR)

### Description of the trajectory groups

**Fig. 2:**
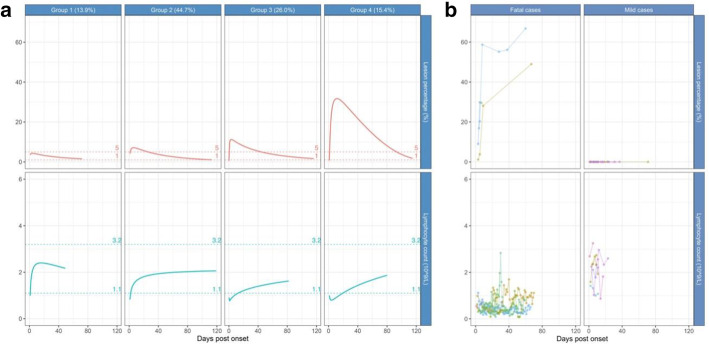
Longitudinal trajectories of lesion percentage (top row) and lymphocyte count (bottom row) among convalescent (N = 257), mild (N = 5), and fatal (N = 7) COVID-19 patients. **a** Estimated longitudinal trajectories for convalescent patients that were categorised into four groups based on group-based multi-trajectory analysis. **b** Individual longitudinal trajectories for mild and fatal cases with the observed data

The model most optimised for fit identified four distinct trajectory groups in terms of pneumonia lesions and lymphocyte counts (Fig. 2a). For lesion percentage, all the four trajectories followed a mountain-like shape of soaring to a peak and then being gradually absorbed. From Groups 1 to 4, both the peak lesion percentage and time from onset to peak increased in steps, suggesting the increasing severity of pneumonia (Table 2). Two main types of lymphocyte count patterns existed. One started at a low level and then rapidly increased, while the other declined for a short time and then slowly recovered. Additional file 1: Table S1 displays the metrics of adequacy and fit for the ultimate trajectory model, all demonstrating that the model was well fit.

**Table 2:** Characteristics of peak lesion percentages by trajectory groups

Variable	Overall (N = 257)	Group 1 (N = 36)	Group 2 (N = 117)	Group 3 (N = 64)	Group 4 (N = 40)	P value
Peak lesion percentage, %	9.3 (3.9, 20.0)	1.9 (0.7, 4.4)	7.2 (3.2, 12.7)	14.2 (8.5, 19.8)	41.4 (34.8, 47.9)	< 0.001
Days from symptom onset to peak lesion percentage	10.0 (7.0, 12.0)	6.5 (4.0, 9.2)	9.0 (6.0, 12.0)	10.0 (8.0, 12.0)	12.0 (10.8, 14.0)	< 0.001

Statistics presented: median (IQR). Statistical test performed: Kruskal-Wallis test

Specifically, in Group 1, the pneumonia pattern was a small hill with the peak lesion percentage at 1.9% (IQR 0.7–4.4), and the time from onset to peak was 6.5 days (IQR 4.0–9.2) (Fig. 2a, Table 2). No serious lymphopenia was observed before lymphocytes soon restored to 2 × 10^9^/L and above after 4 days, indicating a rapid recovery of the immune system. A total of 36 patients (13.9%) were assigned to Group 1.

In Group 2, the peak lesion percentage was 7.2% (IQR 3.2–12.7), and the time from onset to peak was 9.0 days (IQR 6.0–12.0). The lymphocyte count started from around 0.8 × 10^9^/L and then increased to normal. The lymphocytes restored to 2 × 10^9^/L after 63 days, indicating a fairly quick recovery of the immune function. A total of 115 patients (44.7%) were assigned to Group 2.

In Group 3, the peak lesion percentage reached 14.2% (IQR 8.5–19.8), and the time from onset to peak was 10.0 days (IQR 8.0–12.0). Lymphopenia was exacerbated soon after onset as the lymphocyte count further decreased to 0.75 × 10^9^/L after one day and then slowly recovered to normal. A total of 64 patients (26.0%) were assigned to Group 3.

In Group 4, the peak lesion percentage reached as high as 41.4% (IQR 34.8–47.9), and the time from onset to peak was 12.0 days (IQR 10.8–14.0). The lymphocyte count declined for four days to a nadir of 0.80 × 10^9^/L before gradually recovering. A total of 40 patients (15.4%) were assigned to Group 4. Both Groups 3 and 4 did not experience the repletion of lymphocytes to 2 × 10^9^/L at the end of follow-up, which was as long as 80 days.

Among the four groups, there were significant differences in terms of the days from onset to discharge, the lesion percentages right before discharge, the lymphocyte counts right before discharge, and the percentage of patients who recovered from pneumonia (all *P* < 0.001; Additional file 1: Tables S2 and S3).

For the sake of missing enough data to assess the clinical course from admission to post-discharge, mild and fatal cases were not included in the GBMTM analysis. Instead, we manually plotted the trajectories of these two subpopulations for research integrity. The mild cases had zero lesion throughout hospitalisation, and their lymphocyte counts were maintained in the normal range of 1.1–3.2 × 10^9^/L though fluctuated. As for the fatal cases, the lesion percentages increased rapidly to a much higher level than that of Group 4, while the lymphocyte count almost remained below 1 × 10^9^/L (Fig. 2).

### **Disease severity associated with trajectory group membership**

Clinical subtypes indicate overall disease severity. We further explored the associations between the trajectories and the clinical subtypes. For the clinical subtype on admission, patients in Group 1 were mostly moderate cases except one mild case (Table 3). All patients in Group 2 were moderate cases. Most patients in Group 3 were moderate cases but one patient was severe. Group 4 contained most severe or critical cases (83.3%). Clinical subtypes could change as the patients recover or progress to be more severe, so we also investigated the worst clinical subtype during hospitalisation. During hospitalisation, the distribution of clinical subtypes for any of the first three trajectory groups remained fairly stable, except the prevalence of severe or critical cases in Group 4 increased from 12.5% (5/40) to 32.5% (13/40).

**Table 3:** Differences in disease severity between trajectory groups

Characteristics	Group 1 (N = 36)	Group 2 (N = 117)	Group 3 (N = 64)	Group 4 (N = 40)	P value
*Clinical subtype on admission*					< 0.001
Mild	1 (2.8%)	0 (0%)	0 (0%)	0 (0%)	
Moderate	35 (97.2%)	117 (100%)	63 (98.4%)	35 (87.5%)	
Severe	0 (0%)	0 (0%)	1 (1.6%)	4 (10.0%)	
Critical	0 (0%)	0 (0%)	0 (0%)	1 (2.5%)	
*Worst clinical subtype during hospitalisation*					
Moderate	36 (100%)	117 (100%)	62 (96.9%)	27 (67.5%)	
Severe	0 (0%)	0 (0%)	2 (3.1%)	8 (20.0%)	
Critical	0 (0%)	0 (0%)	0 (0%)	5 (12.5%)	

Statistics presented: median (IQR), n (%); Statistical tests performed: Kruskal-Wallis, chi-squared, or Fisher’s exact tests as needed

In short, from Groups 1 to 4, disease severity was increasingly worse, which was consistent from admission to discharge.

### Basic characteristics associated with trajectory group membership

The basic characteristics investigated included age, sex, smoking history, and comorbidity (Table 4). Overall, age, hypertension and diabetes were significantly different between the groups. The median age was 34 years (IQR 30–41), 44 years (IQR 36–60), 56 years (IQR 46–64), and 65 years (IQR 52–69) in Groups 1 to 4, respectively (P < 0.001). Patients in Group 1 were most unlikely to be hypertensive (2.8% compared to higher than 20% in the other groups [P = 0.013]). No patients in Group 1 had diabetes but the prevalence of diabetes in Group 4 was as high as 20% (P = 0.021).

**Table 4:** Basic characteristics by trajectory groups

Characteristics	Group 1 (N = 36)	Group 2 (N = 117)	Group 3 (N = 64)	Group 4 (N = 40)	P value
Age, years (IQR)	34 (30, 41)	44 (36, 60)	56 (46, 64)	65 (52, 69)	< 0.001
Male sex, n (%)	18 (50.0%)	59 (50.4%)	31 (48.4%)	23 (57.5%)	0.832
Smoking, n (%)	4 (11.1%)	7 (6.0%)	5 (7.8%)	4 (10.0%)	0.630
*Symptoms*					
Fever	28 (77.8%)	99 (84.6%)	50 (78.1%)	37 (92.5%)	0.204
Cough	28 (77.8%)	94 (80.3%)	57 (89.1%)	31 (77.5%)	0.346
Diarrhoea	4 (11.1%)	27 (23.1%)	11 (17.2%)	7 (17.5%)	0.406
*Comorbidity*					
Cardiovascular disease	0 (0%)	9 (7.7%)	5 (7.8%)	4 (10.0%)	0.289
Hypertension	1 (2.8%)	30 (25.6%)	15 (23.4%)	13 (32.5%)	0.013
Coronary heart disease	0 (0%)	9 (7.7%)	5 (7.8%)	4 (10.0%)	0.289
Diabetes	0 (0%)	14 (12.0%)	5 (7.8%)	8 (20.0%)	0.021
Chronic obstructive pulmonary disease	0 (0%)	1 (0.9%)	4 (6.2%)	1 (2.5%)	0.100
Cancer	2 (5.6%)	1 (0.9%)	3 (4.7%)	0 (0%)	0.108

Statistics presented: median (IQR), n (%); Statistical tests performed: Kruskal-Wallis, chi-squared, or Fisher’s exact tests as needed

## Discussion

Using GBMTM, for the first time we identified four distinct trajectory groups based on individual time series data of the long-term pneumonia lesion percentage and lymphocyte patterns, providing a comprehensive depiction of the COVID-19 disease course from admission to post-discharge. Regarding lesion percentage, all four trajectories appeared to be positively skewed hill-like curves, and the peak value progressively increased from Groups 1 to 4. For lymphocyte counts, two main types were observed. Groups 1 and 2 started at a low level and then rapidly increased, while Groups 3 and 4 decreased for a short time and then slowly recovered. Patients in the higher order groups were older and more likely to have hypertension and diabetes. Patients in Groups 1 to 4 were characterised with sequentially more severe disease. Furthermore, we found fatal cases were worse than in Group 4 with significantly more pneumonia lesions and lower lymphocyte counts. In contrast, the mild cases demonstrated no pneumonia lesions and mostly normal lymphocyte counts.

Characterising the long-term trajectories enabled us to assess the population heterogeneity over the COVID-19 disease course. Despite the importance of longitudinal evaluation of chest CT and laboratory results as standards of clinical care, only simple and qualitative assessments of the follow-up reports were carried out subjectively in clinical practice. In research areas, a few studies explored the temporal changes of chest CT findings and reported that the lung abnormalities peaked 6–11 days post onset [2, 6, 26, 27], which was consistent with our finding (Table 2). Regarding the laboratory biomarkers, our results were in accordance with Tan’s study [28], which pointed out that in severe patients the lymphocyte number progressively decreased at the progression stage and the peak stage, but increased in the recovery stage. Chen et al. observed that decreased lymphocyte counts recovered to normal in convalescent patients, whereas they maintained low levels among the deceased [5]. A recent meta-analysis demonstrated that alterations of lymphocyte after treatment was a reliable indicator for predicting COVID-19 progression or mortality [29]. These findings were also confirmed by our results.

However, little is known about the different dynamic evolution patterns across population from previous studies. The previous studies usually ignored the heterogeneity between patients by only estimating cross-sectional population-level summaries of biomarkers. Instead, we took a step forward by applying GBMTM to analyse the patient-level time series of pneumonia lesion percentage and lymphocyte count, in which the interrelationships between the two biomarkers, i.e. the pneumonia lesions and lymphocytes, were also captured. Our results of the two biomarkers were consistent with Wang’s research [30], in which the reduction in the peripheral blood lymphocyte level caused by COVID-19 was significantly related to the degree of lung lesions. What’s more, our results further showed that COVID-19 patients were heterogeneous and the convalescent patients could be further divided into four subgroups.

Clustering COVID-19 patients into four trajectory groups could be a better method for understanding the disease severity of various patients and thus be useful for triage for tailored treatment. As shown in the results, the trajectory grouping was a stable indicator of overall disease severity, no matter compared to single measures such as lesion percentage, lymphocyte count, or clinical subtypes. This could be because the trajectory groups can capture the dynamic patterns of several key biomarkers instead of one single biomarker at only one single time point. As shown in Fig. 2a, from Groups 1 to 4, the peak lesion level and time from onset to peak level became increasingly larger, and the time for lymphocyte counts to rebound was gradually longer. The severe or critical cases were mostly in Groups 3 and 4 on admission, and more patients in Groups 3 and 4 developed more severe status during hospitalisation. These findings suggest that patients requiring a long time to resolve pneumonia and restore lymphocyte counts should be assigned to intensified care, especially those with declining lymphocyte counts.

Importantly, we observed that the lymphocyte counts were replenished much earlier than the resolution of lung lesions, indicating immune system restoration precedes lung tissue repair. Lymphocyte counts started to recover before symptom onset among the patients in Groups 1 and 2, and after 1 day and 4 days post-onset for the patients in Groups 3 and 4. The durations for lymphocyte count recovery to the normal level (at least 1.1 × 10^9^/L) were 1 day, 2 days, 13 days, and 19 days in Groups 1 to 4, respectively. However, the durations before pneumonia began to resolve were 6.5, 9, 10, and 12 days in Groups 1 to 4, respectively. In light of these findings, monitoring the trends in lymphocyte alterations and the turning point of recovering lymphocytes may help rapidly identify patients at a high risk of adverse outcomes or who are recovering compared with pneumonia lesion volume changes on CT, which are organic injuries and will lag behind in reflecting the disease progression trend. As a result, optimised treatment could be promptly assigned to help reduce complications and mortality.

The heterogeneity in the longitudinal trajectories is also valuable for optimising post-discharge follow-up strategies, such as choosing proper follow-up intervals for different patients. According to previous studies, more than 80% of patients with COVID-19 have residual lesions on CT right before discharge [2, 31]. In our study, we found that the lesion percentages right before discharge were 0.6%, 2.7%, 6.0%, and 24.6% in Groups 1 to 4, and lymphocyte counts right before discharge were 2.5 × 10^9^/L, 1.9 × 10^9^/L, 1.2 × 10^9^/L, and 1.5 × 10^9^/L in Groups 1 to 4, respectively (Additional file 1: Table S2). This demonstrated that the biomarkers were not fully recovered and the values varied across different COVID-19 patients at discharge, indicating that the follow-up strategy should consider such heterogeneity. For example, the follow-up interval after discharge might be chosen with time 0 as the day of symptom onset. Apart from Group 1, which always had a pneumonia percentage less than 5%, the durations from onset to lesion percentage decreasing to 5% were estimated to be 26, 45, and 93 days in Groups 2 to 4, respectively. The durations from onset to lymphocyte count returning to 1.1 × 10^9^/L were estimated to be 1 day, 2 days, 13 days, and 19 days in Groups 1 to 4, respectively. Using these results, we can adjust the follow-up intervals of different subgroups. In general, patients in Groups 3 and 4 needed a more frequent follow-up plan to obtain more intensive surveillance during their longer convalescence period.

There are several limitations in our study. First, we focused on pneumonia lesion volume and lymphocyte counts for the trajectory analysis and ignored other clinical indicators, for example, leukocyte counts and D-dimers, which might have resulted in losing some pieces of the complete picture of COVID-19’s disease evolution. However, CT imaging and lymphocyte counts are usually among the easiest biomarkers to access and interpret at most hospitals, thus making this study more reproducible. Second, to avoid the transmission risk caused by coughing and droplet formation during testing [32], no data on pulmonary function tests were collected during follow-up to assess the survivors’ recovery of lung function. Third, the entire follow-up period was at least two months for all of the patients. Longer follow-up is expected to depict extended evolution of the disease. Finally, future studies are anticipated to collect more densified data points to predict the trajectory group membership of a patient in an early stage of the disease course, which would be beneficial to guide therapeutic strategies as a patient predicted in a higher order group should undergo more intensive surveillance to prevent undesirable outcomes.

## Conclusions

In conclusion, our study identified four different trajectory groups in terms of pneumonia lesions quantified on CT and lymphocyte counts, an indicator of immune system function, in a cohort of convalescent COVID-19 patients with different characteristics between groups. Generally, in trajectory Groups 1 to 4, the degree of disease severity gradually increased. These distinct trajectory groups suggested that COVID-19 patients are heterogenous and should be further subtyped and treated differently. These findings shed new light on understanding the underexplored course of the disease, highlight the need for clinicians to closely monitor the disease course even after hospital discharge, and provide new perspectives to study COVID-19 patients to generate knowledge for improved prevention, control, and therapeutics.

## Supplementary Information

**Additional file 1.** Additional methods, results, tables and figures.

## Data Availability

The datasets used and/or analysed during the current study are not publicly available as they contain identifiable and personal information of COVID-19 but are available from the corresponding authors on reasonable request.

## Abbreviations

COVID-19: Coronavirus disease 2019
CT: Computed tomography
GBMTM: Group-based multi-trajectory modelling
SPHCC: Shanghai Public Health Clinical Center
IQR: Interquartile range
