# Supplementary material for: Longitudinal trajectories of pneumonia lesions and lymphocyte counts associated with disease severity among convalescent COVID-19 patients: a group-based multi-trajectory analysis
Source: BMC Pulm Med. 2021 Jul 13;21:233. doi: 10.1186/s12890-021-01592-6 (PMC8276845; doi:10.1186/s12890-021-01592-6)
Supplement: Supplementary file 1 — Additional file 1. Additional methods, results, tables and figures. [file 12890_2021_1592_MOESM1_ESM.docx]

**Supplementary materials**

**Longitudinal Trajectories of Pneumonia Lesions and Lymphocyte Counts Associated with Disease Severity among Convalescent COVID-19 Patients: A Group-Based Multi-Trajectory Analysis**

Nannan Shi^1*^, Chao Huang^2,3*^, Qi Zhang^2,3,4,5#^, Chunzi Shi^1^, Fengjun Liu^1^, Fengxiang Song^1^, Qinguo Hou^1^, Jie Shen^1^, Fei Shan^1^, Xiaoming Su^3^, Cheng Liu^2,3^, Zhiyong Zhang^6^, Lei Shi^2,3^, Yuxin Shi^1#^

*Joint first authors, contributed equally

1 Department of Radiology, Shanghai Public Health Clinical Center, Fudan University, Shanghai, China;

2 Shanghai Key Laboratory of Artificial Intelligence for Medical Image and Knowledge Graph, Shanghai, China;

3 Institute of Healthcare Research, Yizhi, Shanghai, China;

4 Shanghai Institute for Advanced Communication and Data Science, Shanghai University, Shanghai, China;

5 School of Communication and Information Engineering, Shanghai University, Shanghai, China;

6 Fudan University, Shanghai, China;

^#^Correspondence to:

Prof Yuxin Shi,

Department of Radiology, Shanghai Public Health Clinical Center, Fudan University, Shanghai 201508, China

[shiyx828288@163.com](mailto:shiyx828288@163.com)

or

Dr. Qi Zhang,

Shanghai Key Laboratory of Artificial Intelligence for Medical Image and Knowledge Graph, Shanghai 200051, China

qizhang.fdu@gmail.com

**Methods**

**Chest CT acquisition and AI-based COVID-19 pneumonia lesion quantification**

SCENARIA 64 CT (Hitachi Medical, Japan), Brilliance 64 CT (Philips, Netherlands), and uCT528 (United Imaging, China) were used with scanning parameters of 120 kV. The low-dose chest smart milliampere (180 mA-400 mA) scanning method was used to reduce the dose. Other CT imaging protocol settings were as follows: rotation time: 0.5 second; collimation: 0.625 mm; pitch: 1.5; and matrix: 512 x 512. Reconstruction was performed with a thickness of 1 mm.

As shown in **Figure S1**, the Quantitative Evaluation System of CT for COVID-19 (YT-CT-Lung, YITU Healthcare Technology Co., Ltd., China) was employed as the CT image quantisation and analysis tool under the supervision of two board-certified radiologists with more than 10 years of experience. The system combined a fully convolutional network with adaptive thresholding and morphological operations for segmentation of lungs and pneumonia lesions(1, 2). External validation with 383 axial CT images from 206 patients had a Dice coefficient of 82.08% for COVID-19 pneumonia lesion segmentation(3). Pneumonia lesion volumes and lung volumes were calculated with the segmentation masks and were used to compute the percentages of lung volumes involved with pneumonia lesions (hereinafter referred to as lesion percentage).

**Steps of the group-based multi-trajectory modeling (GBMTM) and assessment of the model**

GBMTM uses maximum likelihood estimation to estimate both the trajectory shape for each group (modelled as a complex function of the biomarkers and time) and the estimated probabilities of each individual belonging to each trajectory group given their observed data(4). Each individual is then assigned to a specific group based on the highest probability of group membership. The number of trajectory groups and order of the polynomials of time function are not actually known priori and are clarified using systematic model search following the guides in Nagin et al.(4-6) 112122 . In our analyses, both lesion percentage and lymphocyte count were modelled assuming a censored normal distribution and identity link. The trajectories were assumed to follow a quadratic function of log-transformed days because we found that this setting coincided well with the observed data and was in line with the knowledge drawn from clinical practise for COVID-19. GBMTM analyses were conducted with the traj package using Stata version 14.1 (StataCorp, College Station, TX, USA).

The first GBMTM step was separately estimating the trajectory models with varying numbers of groups for each biomarker to identify the proper groups of distinct trajectories composing the overall population. The second step was varying the order of the trajectory polynomials of time based on professional knowledge about the variables to select the model best optimised to fit and parsimony using the Bayesian information criterion.

We assessed our final GBMTM model in terms of adequacy and fit of the trajectories using the following established metrics: (1) Group average posterior probability: for each group, we computed the average probability for individuals assigned to the group. (2) Odds ratio for correct classification: the odds ratio of probability of being assigned to the trajectory group in comparison to the estimated population-level group distribution. (3) Estimated group distribution based on using maximum probability assignment rule. (4) Estimated group distribution based on initial model. (5) Entropy: an indicator of discrepancy between trajectory groups(4, 7-9).

**Results**

**Assessment metrics of the modelled trajectories**

First, the average posterior probabilities for each group were all greater than 0.9, which was much higher than the criteria of 0.7. Second, the odds ratios for correct classifications were also much greater than 5 in all of the groups. Third, the estimated group distribution using the maximum probability assignment rule was pretty close to that based on the model. Finally, the overall entropy was 0.864, which was larger than 0.8 as required. Overall, the assessment results showed that the model had a very good fit and could discriminate the trajectory groups.

**Extended quantitative comparison between the four trajectory groups**

More metrics were derived to quantitatively compare the four trajectories. First, CT scans and biochemical tests right before discharge were extracted for each individual and analysed (**Table S2**). In Groups 1 to 4, the days from onset to discharge were 20 (IQR 16~26), 20 (IQR 17~25), 21 (IQR 18~26), and 29 days (24~34) (P < 0.001). The lesion percentages right before discharge were 0.6% (IQR 0.1~1.5), 2.7% (IQR 0.8~5.7), 6.0% (IQR 2.7~10.5), and 24.6% (IQR 17.0~28.8) (P < 0.001). The lymphocyte counts right before discharge were 2.5 × 10^9^/L (IQR 2.2~2.9), 1.9 × 10^9^/L (IQR 1.6~2.1), 1.2 × 10^9^/L (IQR 1.1~1.5), and 1.5 × 10^9^/L (IQR 1.1~1.7) (P < 0.001). It was noted that for all of the groups, the average time from either the selected CT scans or biochemical tests to discharge was 2~3 days and there was no significant difference between the groups (both P > 0.05; **Table S2**), suggesting that the lesion percentages and lymphocyte counts mentioned were appropriate to be compared between the groups.

Furthermore, we compared the pneumonia absorption trends between the estimated trajectories (**Table S3**). Briefly, the percentage of patients who recovered from pneumonia 30 days (or 60 days) after symptom onset (or after discharge) was always the highest in Group 1, followed by Groups 2, 3, and 4, no matter whether the lesion percentage threshold of substantial pneumonia absorption was set at 5% or 1% (all P < 0.001). Based on the estimated trajectories, we noted that Group 1 had pneumonia lower than 5% all of the time (**Figure 2A**). The durations from onset to lesion percentage decreasing to 5% were estimated to be 26, 45, and 93 days in Groups 2 to 4, respectively. For the four estimated trajectories, the lesion percentages at the end of follow-up approached 1%. Moreover, the durations from onset to lymphocyte count recovering to normal were estimated to be 1 day, 2 days, 13 days, and 19 days in Groups 1 to 4, respectively.

**Supplemental Tables**

Table S1. Metrics of adequacy and fit of trajectory model

| Trajectory group | Group average posterior probability | Odds ratio for correct classification | Estimated group distribution based on using the maximum probability assignment rule | Estimated group distribution based on the initial model | Entropy |
| --- | --- | --- | --- | --- | --- |
| 1 | 0.900 | 64.88 | 0.140 | 0.139 | 0.864 |
| 2 | 0.909 | 22.29 | 0.455 | 0.447 |  |
| 3 | 0.920 | 43.97 | 0.249 | 0.260 |  |
| 4 | 0.985 | 431.29 | 0.156 | 0.154 |  |

Criteria for good model fit: (1) average posterior probability greater than 0.7 in each group; (2) odds ratio of correct classification greater than 5 in each group; (3) close correspondence between the estimated group distribution based on using posterior probabilities, the maximum probability assignment rule, and the estimated group distribution from the initial model; and (4) an entropy value greater than 0.8.

Table S2. Lesion percentage and lymphocyte count at discharge by trajectory group

| **Variable** | **Overall**  **(N = 257)** | **Group 1**  **(N = 36)** | **Group 2**  **(N = 117)** | **Group 3**  **(N = 64)** | **Group 4**  **(N = 40)** | **P value** |
| --- | --- | --- | --- | --- | --- | --- |
| **Days from onset to discharge** | 21 (17, 28) | 20 (16, 26) | 20 (17, 25) | 21 (18, 26) | 29 (24, 34) | < 0.001 |
| **Lesion percentage, %** | 4.1 (1.0, 9.8) | 0.6 (0.1, 1.5) | 2.7 (0.8, 5.7) | 6.0 (2.7, 10.5) | 24.6 (17.0, 28.8) | < 0.001 |
| **Days from last CT scan in hospital to discharge** | 2 (1, 3) | 3 (1, 4) | 2 (1, 4) | 2 (1, 3) | 3 (2, 3) | 0.063 |
| **Lymphocyte count, 10^9^/L** | 1.7 (1.4, 2.1) | 2.5 (2.2, 2.9) | 1.9 (1.6, 2.1) | 1.2 (1.1, 1.5) | 1.5 (1.1, 1.7) | < 0.001 |
| **Days from last biochemical test in hospital to discharge** | 3 (2, 4) | 3 (2, 4) | 3 (2, 4) | 3 (2, 4) | 3 (2, 4) | 0.157 |

Note: Statistics presented: median (IQR). Statistical test performed: Kruskal-Wallis test.

Table S3. Number of patients with most pneumonia lesions absorbed after symptom onset and after discharge

| **Variable** | **Overall**  **(N = 257)** | **Group 1**  **(N = 36)** | **Group 2**  **(N = 117)** | **Group 3**  **(N = 64)** | **Group 4**  **(N = 40)** | **P value** |
| --- | --- | --- | --- | --- | --- | --- |
| **Days post-symptom onset** |  |  |  |  |  |  |
| Lesion percentage <= 5% at 30 days | 158 (61.5%) | 36 (100%) | 89 (76.1%) | 33 (51.6%) | 0 (0%) | < 0.001 |
| Lesion percentage <= 5% at 60 days | 213 (82.9%) | 36 (100%) | 112 (95.7%) | 54 (84.4%) | 11 (27.5%) | < 0.001 |
| Lesion percentage <= 1% at 30 days | 89 (34.6%) | 26 (72.2%) | 53 (45.3%) | 10 (15.6%) | 0 (0%) | < 0.001 |
| Lesion percentage <= 1% at 60 days | 160 (62.3%) | 34 (94.4%) | 90 (76.9%) | 33 (51.6%) | 3 (7.5%) | < 0.001 |
| **Days post-discharge** |  |  |  |  |  |  |
| Lesion percentage <= 5% at 30 days | 208 (80.9%) | 36 (100.0%) | 109 (93.2%) | 49 (76.6%) | 14 (35.0%) | < 0.001 |
| Lesion percentage <= 5% at 60 days | 219 (85.2%) | 36 (100.0%) | 111 (94.9%) | 55 (85.9%) | 17 (42.5%) | < 0.001 |
| Lesion percentage <= 1% at 30 days | 146 (56.8%) | 34 (94.4%) | 84 (71.8%) | 26 (40.6%) | 2 (5.0%) | < 0.001 |
| Lesion percentage <= 1% at 60 days | 172 (66.9%) | 34 (94.4%) | 94 (80.3%) | 38 (59.4%) | 6 (15.0%) | < 0.001 |

Note: Statistics presented: n (%). Statistical test performed: chi-squared test.

**Supplemental Figures**


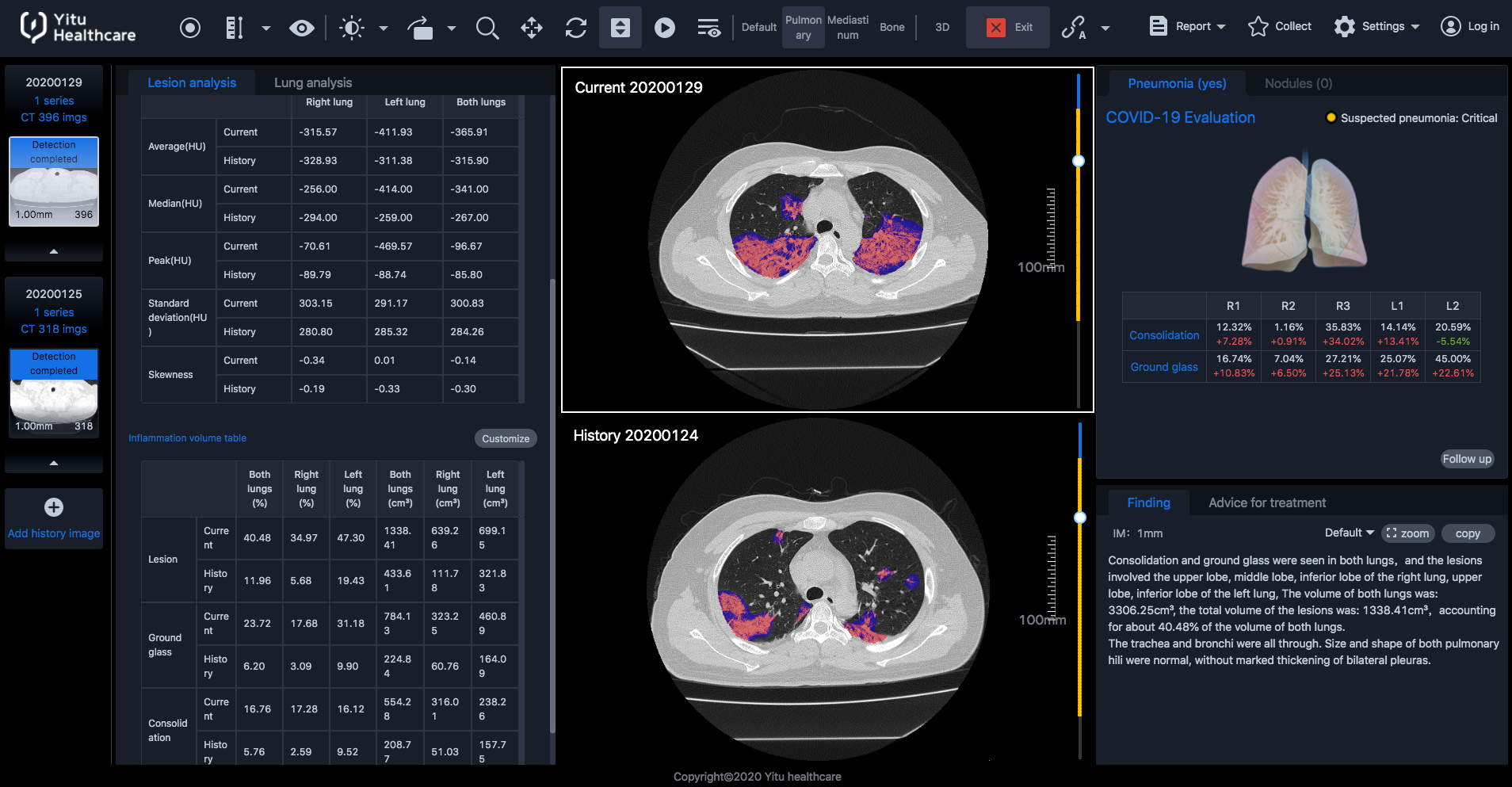


Figure S1. The AI-based system used for automatic CT image quantisation.

**References in the supplementary materials**

1. Falk T, Mai D, Bensch R, Çiçek Ö, Abdulkadir A, Marrakchi Y et al. U-Net: Deep learning for cell counting, detection, and morphometry. Nat Methods 2019;16:67-70

2. Wang S, Zhou M, Liu Z, Liu Z, Gu D, Zang Y et al. Central focused convolutional neural networks: Developing a data-driven model for lung nodule segmentation. Med Image Anal 2017;40:172-183

3. Liu F, Zhang Q, Huang C, Shi C, Wang L, Shi N et al. CT quantification of pneumonia lesions in early days predicts progression to severe illness in a cohort of COVID-19 patients. Theranostics 2020;10:5613-5622

4. Nagin DS, Jones BL, Passos VL, Tremblay RE. Group-based multi-trajectory modeling. Stat Methods Med Res 2018;27:2015-2023

5. Nagin D. Group-based modeling of development. Cambridge: Harvard University Press; 2005.

6. Wang C, Huang P, Wang L, Zhujing S, Lin B, Wang Q et al. Temporal changes of COVID-19 pneumonia by mass evaluation using CT: A retrospective multi-center study. Annals of Translational Medicine 2020;8

7. Mody A, Eshun-Wilson I, Sikombe K, Schwartz SR, Beres LK, Simbeza S et al. Longitudinal engagement trajectories and risk of death among new ART starters in Zambia: A group-based multi-trajectory analysis. Plos Med 2019;16:e1002959

8. Nagin DS, Odgers CL. Group-based trajectory modeling in clinical research. Annu Rev Clin Psychol 2010;6:109-138

9. Klijn SL, Weijenberg MP, Lemmens P, van den Brandt PA, Lima PV. Introducing the fit-criteria assessment plot - a visualisation tool to assist class enumeration in group-based trajectory modelling. Stat Methods Med Res 2017;26:2424-2436
